# Healthcare Expenditure on Atrial Fibrillation in the United States: The Medical Expenditure Panel Survey 2016 to 2021

**DOI:** 10.1016/j.jacadv.2025.101716

**Published:** 2025-04-25

**Authors:** Claudia See, Scott Grubman, Nisarg Shah, Jiun-Ruey Hu, Michael Nanna, James V. Freeman, Karthik Murugiah

**Affiliations:** a Division of General Internal Medicine, University of California, San Francisco, California, USA; b Division of General Internal Medicine, The Mount Sinai Hospital, New York, New York, USA; c Yale School of Medicine, New Haven, Connecticut, USA; d Department of Cardiology, Smidt Heart Institute, Cedars-Sinai Medical Center, Los Angeles, California, USA; e Section of Cardiovascular Medicine, Department of Internal Medicine, Yale School of Medicine, New Haven, Connecticut, USA; f Center for Outcomes Research and Evaluation, Yale New Haven Health Services Corporation, New Haven, Connecticut, USA

**Keywords:** atrial fibrillation, comorbidities, expenditures, socioeconomic status

## Abstract

**Background:**

Atrial fibrillation (AF) prevalence is increasing, and management is evolving. This study builds on prior studies focusing on AF healthcare expenditures.

**Objectives:**

The purpose of this study was to provide a contemporary nationally representative assessment of AF and atrial flutter expenditures in the United States.

**Methods:**

Using Medical Expenditure Panel Survey 2016 to 2021 data, we identified individuals with AF or atrial flutter using International Classification of Disease-10 codes and reported total and categorized expenditures. Using 2-part and gamma regression models, respectively, we estimated the incremental AF expenditures for the entire population and for individuals with common coexisting comorbidities. Among AF individuals, we identified characteristics associated with higher expenditures.

**Results:**

Of a weighted surveyed population of 248,067,064 adults, 3,564,763 (1.4%) had AF, with a mean age 71.9 ± 10.6 years, and 45.7% were female. The mean unadjusted annual total healthcare expenditure was $25,451 ± $1,100 ($9,254 ± $82 without AF). Highest spending categories were inpatient visits ($7,975 ± $733) and prescriptions ($6,505 ± $372). AF expenditures increased over the study period by 11.1%. After adjustment, the incremental annual expenditure attributable to AF was $6,185 per person. Incremental AF expenditures were highest for those with cancer at $12,052 ($4,476-$19,627), while AF did not significantly increase expenditures in heart failure at $30 (−$7,660 to $7,716). Cancer, modified Charlson Comorbidity Index of 1 or ≥2, chronic kidney disease, type 2 diabetes mellitus, chronic obstructive pulmonary disease, atherosclerotic cardiovascular disease, poor income level, bachelor’s degree, and later survey year were associated with higher expenditures.

**Conclusions:**

AF is associated with substantial and increasing healthcare expenditures. With changing screening and management, expenditures need periodic reassessments.

Atrial fibrillation (AF) is the most common cardiac arrhythmia, affecting between 2.7 and 6.1 million people in the U.S.^1^ Over time, with an aging population and potentially increased detection due to improved and more widespread monitoring devices, the prevalence of AF has been rising, and the prevalence is estimated to be 12.1 million by 2030.^2^

AF is associated with significant morbidity, including hospitalizations and risk of cardiovascular and cerebrovascular events.^3^^,^^4^ Given these characteristics, AF is associated with high healthcare expenditures.^5^^,^^6^ The management of AF has been evolving in recent times, with increased use of antiarrhythmics and procedures to maintain sinus rhythm as well as increased use of direct-acting oral anticoagulants (DOACs) and left atrial appendage occlusion for stroke prevention, which can affect overall expenditures. However, contemporary studies of U.S. national healthcare expenditures for AF care have been lacking. Furthermore, AF frequently co-exists with other important chronic comorbidities such as heart failure (HF), atherosclerotic cardiovascular disease (ASCVD), chronic kidney disease (CKD), chronic obstructive pulmonary disease (COPD), cancer, type 2 diabetes mellitus (T2DM), and hypertension (HTN), all of which may complicate their management.^7^, ^8^, ^9^ How expenditures of these conditions are influenced by co-existent AF has not been well evaluated.

Accordingly, the aim of this study was to report current nationally representative healthcare expenditures and recent trends for individuals with AF (categorizing expenditures across medical service categories such as hospitalizations and prescriptions), estimate the incremental expenditures attributable to AF, and identify the characteristics of individuals with AF who experience higher expenditures.

## Methods

### Data sources

The Medical Expenditure Panel Survey (MEPS) is an annual cross-sectional survey of U.S. families and individuals by the Agency of Healthcare Research and Quality (AHRQ). It is the largest all-payer nationally representative annual cross-sectional survey of medical expenditures of the U.S. civilian, noninstitutionalized population.^10^ MEPS collects data on demographic characteristics, health conditions, health status, use of medical services, charges and source of payments, access to care, satisfaction with care, health insurance coverage, income, and employment. Overall response rates in the years 2016 to 2021 were approximately 22% to 46%.^11^

The panel design of the survey includes 5 rounds of interviews covering 2 full calendar years. Every year, a new panel of about 15,000 sample households is selected. The set of households selected for each panel is a subsample of households participating in the previous year's National Health Interview Survey conducted by the National Center for Health Statistics.

Data from the MEPS Household Component Full-Year Consolidated Data Files and Medical Conditions Files were pooled across the years 2016 to 2021 and then merged using 2 variables—patient identifier variable “DUPERSID” and survey “Year.” The study was limited to 2016 and after because the International Classification of Disease (ICD)-9 codes used in MEPS data before 2016 were limited to three digits for confidentiality reasons and could not distinguish AF from other cardiac arrhythmias. For 2016 and 2017, the 2-digit panel number from the variable “PANEL” was added in front of all DUPERSIDs as per the recommendation of AHRQ^12^ to be consistent across all survey years. Families and individuals were assigned weights based on demographic proportions in the overall U.S. population.^13^ The National Health Interview Survey sample design underwent several changes between 2016 and 2021, such as discontinuation of oversampling of Asian, Black, and Hispanic minority groups^14^ in favor of oversampling of Veterans and middle-sized states.^15^^,^^16^ To compensate for low response rates during the COVID-19 pandemic, MEPS extended the duration of its survey panels by 1 year.^11^

As all data and materials are de-identified and publicly available, this study was exempt from institutional review board approval by Yale University.

### Study population

The study population included all adults aged 18 years or older. AF and atrial flutter were identified using the ICD-10 code “I48.” Similarly, the comorbidities ASCVD, HF, HTN, CKD, COPD, cancer, and T2DM were identified using appropriate ICD-10 codes (Supplemental Table 1). The weighted population size was estimated as the average annual population size for the pooled period, based on the recommendation from AHRQ on pooling.^17^

### Variables and Preprocessing

Age was categorized into 18 to 44, 45 to 64, 65 to 84, and ≥85 years old, and sex as male or female. Race/ethnicity was categorized as non-Hispanic White, Hispanic, non-Hispanic Black, and non-Hispanic Asian and other/mixed race. Marital status was categorized as married, never married, and divorced/widowed/separated. Insurance status was categorized as private, Medicare, other public, or uninsured. Education was categorized as no degree, general educational development or high school graduate, bachelor's degree, master's or doctorate degree, and other degrees. Individuals were classified as living in one of 4 census regions: Northeast, Midwest, South, or West.^18^ Family income was categorized as poor (families with income less than or equal to the federal poverty line [FPL]), near-poor (100%-125% of the FPL), low income (125%-200% of the FPL), middle income (200%-400% of the FPL), and high income (>400% of the FPL) per the MEPS poverty status variable. In the multivariable models, survey year was treated as a continuous variable. The Stata “charlson” package was used to calculate the Charlson Comorbidity Index (CCI) score.^19^ A higher score represents more comorbid conditions.

Total medical expenditure was defined as the sum of direct payments for care across 8 medical service categories: inpatient hospitalization stays, outpatient and office-based visits, prescription medication, emergency department visits, dental visits, home healthcare, and others (including dental and vision expenditures). Payments were combined across 10 payers such as private insurance, Medicare, Medicaid, Tricare, out-of-pocket, and other. All expenditures and incomes in our analyses were inflation adjusted to year 2021 using the Consumer Price Index. Home health expenditures were derived by summed home health agency and home health nonagency expenditure MEPS variables. Other expenditures were derived by summed dental, vision, and “other” expenditure MEPS variables.

Demographic variable data reported as missing in the MEPS database (ie, cannot be computed, do not know, refused, and inapplicable) were excluded from the analysis. In addition, MEPS compensates for missing medical expenditure data through a weighted hot-deck imputation approach, which uses survey responses and weights to impute missing data, preserving sample sizes and reducing nonresponse bias.^20^

### Statistical methods

Continuous variables were presented as both mean ± SD and median (IQR). Categorical variables were presented as proportions. Continuous data were compared with the Wilcoxon rank-sum test (as they did not follow a normal distribution), and categorical data were compared with chi-squared tests. A 2-sided *P* value of <0.05 was used for statistical significance. MEPS survey weights were applied using the “svy” function in Stata. Statistical analysis was conducted using Stata Statistical Software: Release 18 (Stata Corp.).

### Modeling healthcare expenditure

For expenditure data which are typically right-skewed,^21^ a 2-part model was used—the first model is a logistic or probabilistic (probit) regression model which estimates the probability of zero vs positive expenditures, and the second model is conditional on having a positive annual healthcare expenditure, such as a generalized linear model with gamma distribution and a logarithmic-link function that estimates the average expenditure per capita.^22^^,^^23^

First, a 2-part regression model was created using the entire pooled population with and without AF to estimate incremental total healthcare expenditure attributable to AF. Covariates included in multivariable adjustment were AF, age, sex, race/ethnicity, survey year, CCI, poverty level, education status, marital status, geographic region, and insurance status. National estimates of AF expenditure were extrapolated by multiplying the incremental expenditure per person by the survey weighted number of individuals with AF in the U.S. in our study; second, incremental expenditures attributable to AF within subpopulations of individuals with 7 other common chronic comorbidities of ASCVD, HF, HTN, CKD, COPD, cancer, and T2DM were estimated by creating a single gamma regression model for each subpopulation and calculating the average marginal effect with 95% CIs. Gamma models were used instead of 2-part models because of the low prevalence of individuals with no expenditures within the comorbidity subpopulations. Covariates included in multivariable adjustment were AF, age, sex, race/ethnicity, survey year, modified CCI, individual comorbidities, poverty level, education status, marital status, geographic region, and insurance status. For these gamma regression models, a “modified” CCI was used by excluding the 7 comorbidities of ASCVD, HF, HTN, CKD, COPD, cancer, and T2DM from the CCI calculation,^24^ resulting in 3 final score categories of 0, 1, and ≥2. This was done to enable inputting these comorbidities as separate covariates into the model to study their individual effects.

Third, individual characteristics associated with increased total healthcare expenditures were determined by creating a gamma regression model only for individuals with AF and calculating ORs with 95% CI. For this gamma regression model, the “modified” CCI covariate was also used.

## Results

### Study population characteristics

The average annual surveyed population was 22,911 U.S. adults aged ≥18 years. Of these, 98.6% had complete data (n = 22,583), equivalent to a survey weighted population of 248,067,064 adults. Within this population with complete data, 1,902 survey respondents had AF, equivalent to a survey weighted population of 3,564,763 adults and an overall AF prevalence of 1.4%.

Table 1 shows demographics, comorbidities, and total expenditures by category for the overall population and for those with and without AF.

**Table 1:** Demographics, Comorbidities, and Annual Expenditures of Individuals With and Without AF From 2016 to 2021

	All	AF	Non-AF
Average annual weighted population, N^a^	248,067,064	3,564,763	244,502,302
Average annual sample size, n^a^	22,583	317	22,266
Age categories			
18-44 y	45.8	2.4	46.4
45-64 y	33.1	19.3	33.3
65-84 y	18.8	64.0	18.2
≥85 y	2.3	14.3	2.2
Sex			
Female	51.7	45.7	51.8
Male	48.3	54.3	48.2
Race			
Non-Hispanic White	62.7	89.0	62.3
Hispanic	16.4	3.3	16.6
Non-Hispanic Black	11.9	4.6	12.0
Non-Hispanic Asian and other/mixed race	9.1	3.2	9.1
Marital status (%)			
Married	51.7	59.7	51.6
Widowed/divorced/separated	19.4	34.5	19.2
Never married	28.9	5.9	29.2
Education level			
GED/High school diploma	45.0	50.7	44.9
Bachelor's degree	20.9	14.6	21.0
Master/Doctorate	12.3	19.4	12.2
Other degree	10.1	9.3	10.1
No degree	11.8	6.1	11.9
Census region			
Northeast	17.5	21.4	17.4
Midwest	20.8	25.5	20.8
South	37.9	37.3	38.0
West	23.7	15.9	23.9
Household income level			
Poor/Negative	10.4	7.5	10.5
Near poor	3.7	3.4	3.7
Low income	11.9	13.9	11.9
Middle income	28.5	28.0	28.5
High income	45.4	47.2	45.4
Insurance status			
Private	58.7	16.8	59.3
Medicare	12.5	78.2	12.7
Other public	7.6	4.3	7.7
Uninsured	21.1	0.7	20.2
CCI			
0	83.4	50.6	83.9
1	11.0	26.3	10.8
≥2	5.6	23.0	5.3
Comorbidities			
HTN	32.8	67.9	32.1
T2DM	13.3	22.2	13.1
ASCVD	5.6	34.7	5.2
Cancer	3.5	12.6	3.4
COPD	2.3	9.5	2.2
HF	0.7	7.0	0.6
CKD	0.6	2.1	0.6
Survey year			
2016	16.4	15.3	16.4
2017	16.6	14.4	16.6
2018	16.7	15.9	16.7
2019	16.7	18.2	16.7
2020	16.8	16.4	16.8
2021	16.8	19.8	16.8
Annual unadjusted expenditures^b^			
Total	$7,392 ± $67	$25,451 ± $1,100	$7,129 ± $66
Inpatient	$1,598 ± $33	$7,975 ± $733	$1,506 ± $32
Prescription	$1,822 ± $34	$6,505 ± $372	$1,753 ± $34
Office based	$1,918 ± $24	$4,716 ± $247	$1,877 ± $24
Outpatient	$801 ± $19	$2,867 ± $314	$771 ± $19
Home health^c^	$372 ± $15	$1,345 ± $138	$357 ± $15
ED	$246 ± $4	$715 ± $90	$239 ± $4
Other^d^	$637 ± $6	$1,329 ± $66	$626 ± $7
Annual unadjusted expenditures^b^			
Total	$1,696 ($304-$6,258)	$13,570 ($6,859-$29,708)	$1,616 ($292-$5,979)
Inpatient	$0 ($0-$0)	$0 ($0-$5,684)	$0 ($0-$0)
Prescription	$71 ($0-$698)	$3,223 ($885-$6,626)	$65 ($0-$649)
Office based	$444 ($40-$1,608)	$2,416 ($1,018-$4,909)	$430 ($0-$1,561)
Outpatient	$0 ($0-$0)	$150 ($0-$1,856)	$0 ($0-$0)
Home health^c^	$0 ($0-$0)	$0 ($0-$0)	$0 ($0-$0)
ED	$0 ($0-$0)	$0 ($0-$443)	$0 ($0-$0)
Other^d^	$123 ($0-$559)	$504 ($88-$1,595)	$119 ($0-$550)

Values are %, mean ± SD, or median (IQR). All variables had *P* < 0.001.

a Average annual population sizes are calculated by dividing each total population over 2016 to 2021 by 6 years and rounding up to the nearest whole integer.

b Expenditures are reported in inflation-adjusted 2021 U.S. dollars.

c Home health expenditures are summed as home health agency and home health nonagency expenditure MEPS variables.

d Other expenditures are summed as dental, vision, and other expenditure MEPS variables.

Among individuals with AF, the mean age was 71.9 ± 10.6 years, 45.7% were female, and 89.0% were non-Hispanic White. The median CCI was 1 (IQR: 0-1). Among individuals without AF, the mean age was 47.5 ± 18.2 years, 51.8% were female, and 62.3% were non-Hispanic White. The median CCI was 0 (IQR: 0-0) for individuals without AF. The top chronic comorbidities among individuals with AF (compared with individuals without AF) were HTN (prevalence of 67.9% in those with AF vs 32.1% among those without), T2DM (22.2% vs 13.1%), ASCVD (34.7% vs 5.2%), cancer (12.6% vs 3.4%), COPD (9.5% vs 2.2%), HF (7.0% vs 0.6%), and CKD (2.1% vs 0.6%) (all *P* < 0.001).

### Healthcare expenditures with AF

For individuals with AF, the average unadjusted annual total healthcare expenditure in terms of 2021 inflation-adjusted dollars was $25,451 ± $1,100 (Table 1) compared with $9,254 ± $82 for individuals without AF (*P* < 0.001). For comparison, the average unadjusted annual total healthcare expenditures among individuals with other chronic diseases—CKD, HF, cancer, ASCVD, COPD, T2DM, and HTN—were $46,524 ± $3,129, $33,753 ± $2,026, $25,212 ± $998, $23,096 ± $530, $21,725 ± $675, $17,125 ± $337, and $13,622 ± $207, respectively. Median unadjusted annual total healthcare expenditure for individuals with AF was $13,570 ($6,859-$29,708), compared with $1,616 ($292-$5,979) for individuals without AF (Table 1).

**Figure 1:**
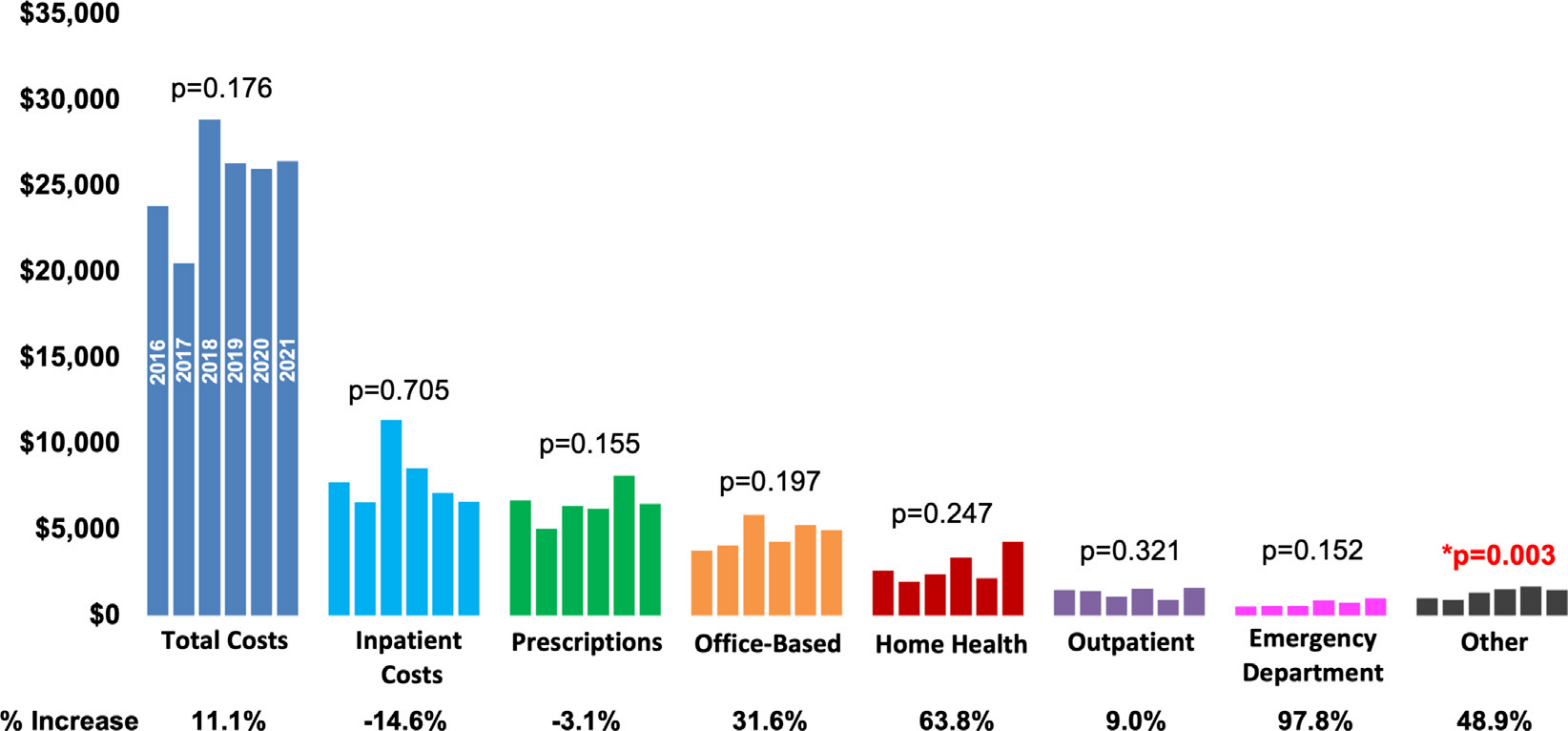
Trends in Healthcare Expenditures Over Time for Individuals With AF This bar graph shows average unadjusted annual total healthcare expenditures for individuals with AF by medical service category by year from 2016 to 2021 with *P* value for significance of trend. ∗Denotes significant *P* value < 0.05.

The highest average expenditures among service categories in AF were inpatient ($7,975 ± $733), prescriptions ($6,505 ± $372), and office-based visits ($4,716 ± $247). Annual expenditures for individuals with AF were higher than those without AF across all service categories. Trends in average unadjusted annual expenditure among individuals with AF over 2016 to 2021 are shown in Figure 1. There was no increasing trend in expenditures across major expenditure categories.

After adjusting for demographics, comorbidities, and survey year, the incremental average annual healthcare expenditure attributable to AF estimated from the 2-part regression model was $6,185 per person (95% CI: $5,082-$7,288) (Supplemental Appendix File 1 for the 2-part model output). With a survey-weighted AF population of 3,564,763 as shown in Table 1, this translates to an overall national healthcare expenditure of $22.1 billion per year.

Incremental expenditures attributable to AF within subpopulations of individuals with the 7 other common chronic comorbidities of cancer, ASCVD, COPD, and HF are shown in Table 2. From largest to smallest, the average marginal effects (95% CI) of having AF in addition to cancer, COPD, T2DM, ASCVD, and HTN were $12,070 ($4,439-$19,700), $8,463 ($3,149-$13,778), $7,918 ($4,837-$10,999), $7,699 ($4,512-$10,886), and $6,665 ($5,084-$8,246), respectively. CKD and HF did not significantly impact AF expenditures with average marginal effects of $3,003 (−$11,490 to $17,496) and $103 (−$7,617 to $7,823), respectively (Central Illustration).

**Table 2:** Incremental Expenditures With AF Among Individuals With Other Common Chronic Comorbidities From Gamma Regression

Subpopulation	Average Annual Sample Size, n^a^	Average Annual Weighted Population, N^a^	AF Prevalence	Incremental Expenditure With AF (95% CI)
Cancer^b^	815	8,706,310	5.1%	$12,070 ($4,439-$19,700)
COPD	581	5,705,185	5.9%	$8,463 ($3,149-$13,778)
T2DM	2,630	24,921,007	2.8%	$7,918 ($4,837-$10,999)
ASCVD	1,389	13,846,252	8.9%	$7,699 ($4,512-$10,886)
HTN	6,101	61,658,796	3.6%	$6,665 ($5,084-$8,246)
CKD	124	1,176,421	6.5%	$3,003 (−$11,490 to $17,496)
HF	176	1,640,324	15.3%	$103 (−$7,617 to $7,823)

Values are average marginal effect (95% CI) of having AF from a gamma regression of individuals with a given comorbidity. Expenditures are reported in inflation-adjusted 2021 U.S. dollars.

AF = atrial fibrillation; ASCVD = atherosclerotic cardiovascular disease; CKD = chronic kidney disease; COPD = chronic obstructive pulmonary disease; HF = heart failure; HTN = hypertension; T2DM = type 2 diabetes mellitus.

a Average annual population sizes are calculated by dividing each total population over 2016 to 2021 by 6 years and rounding up to the nearest whole integer.

b Due to the low sample size in subcategories, the insurance status variable was excluded from the gamma regression model for individuals with cancer to enable convergence.

**Central Illustration:**
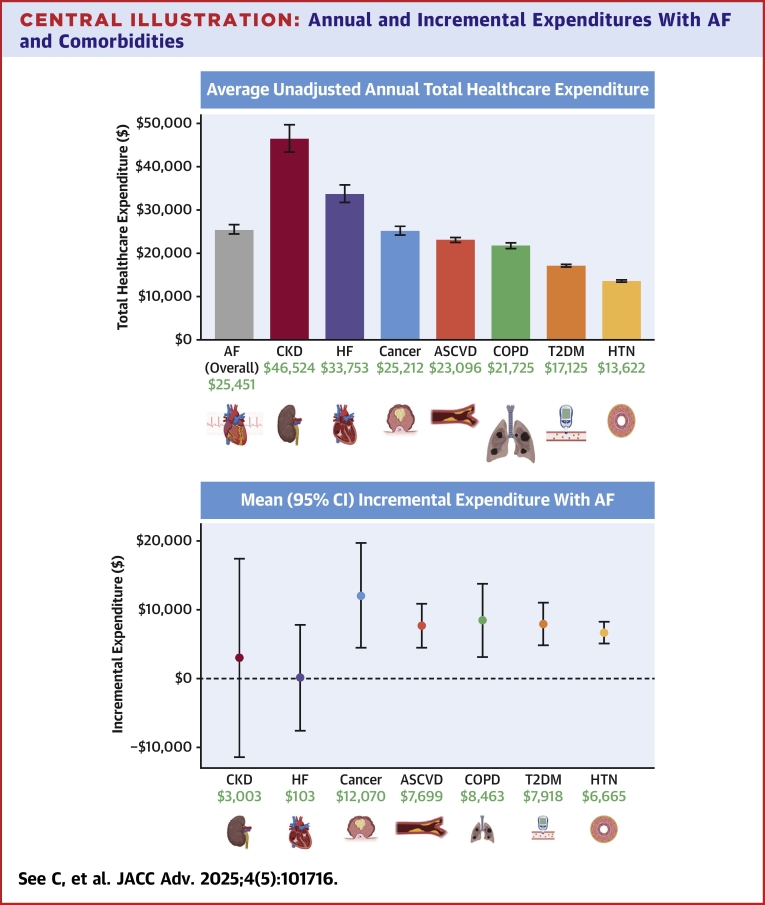
Annual and Incremental Expenditures With AF and Comorbidities The left graph shows the overall average unadjusted annual total healthcare expenditures, independent of AF, for AF and 7 common comorbidities of AF (CKD, HF, cancer, ASCVD, COPD, T2DM, and HF). The right graph shows incremental expenditures of AF among individuals with the 7 comorbidities. Made with BioRender.com. AF = atrial fibrillation; ASCVD = atherosclerotic cardiovascular disease; CKD = chronic kidney disease; COPD = chronic obstructive pulmonary disease; HF = heart failure; HTN = hypertension; T2DM = type 2 diabetes mellitus.

Variables associated with increased total healthcare expenditure among individuals with AF are shown in Table 3. Listing by OR magnitude, higher expenditures were associated with a diagnosis of cancer (OR: 1.84 [95% CI: 1.39-2.43], *P* < 0.001), modified CCI of ≥2 (OR: 1.83 [95% CI: 1.24-2.69], *P* = 0.002) and 1 (OR: 1.30 [95% CI: 1.10-1.54], *P* = 0.003), CKD (OR: 1.74 [95% CI: 1.31-2.32], *P* < 0.001), T2DM (OR: 1.45 [95% CI 1.23-1.71], *P* < 0.001), COPD (OR: 1.38 [95% CI: 1.07-1.78], *P* = 0.014), ASCVD (OR: 1.36 [95% CI: 1.17-1.58], *P* < 0.001), poor (OR: 1.36 [95% CI: 1.02-1.83], *P* = 0.038), bachelor's degree (OR: 1.32 [95% CI: 1.02-1.69], *P* = 0.034), and later survey year (OR: 1.08 [95% CI: 1.03-1.13], *P* = 0.001).

**Table 3:** Factors Associated With Increased Total Healthcare Expenditures Among Individuals With AF From Gamma Regression

	OR (95% CI)	*P* Value
Age categories		
18-44 y (Ref)		
45-64 y	0.59 (0.23-1.51)	0.273
65-84 y	0.63 (0.16-2.44)	0.506
≥85 y	0.72 (0.18-2.80)	0.634
Sex		
Male (Ref)		
Female	0.96 (0.83-1.11)	0.599
Survey year	1.08 (1.03-1.13)	0.001
Race/ethnicity		
Non-Hispanic White (Ref)		
Hispanic	0.97 (0.72-1.31)	0.843
Non-Hispanic Black	0.81 (0.59-1.10)	0.178
Non-Hispanic Asian and other/mixed	1.14 (0.66-1.95)	0.639
Modified CCI		
0 (Ref)		
1	1.30 (1.10-1.54)	0.003
≥2	1.83 (1.24-2.69)	0.002
Comorbidities		
Cancer	1.84 (1.39-2.43)	<0.001
CKD	1.74 (1.31-2.32)	<0.001
T2DM	1.45 (1.23-1.71)	<0.001
COPD	1.38 (1.07-1.78)	0.014
ASCVD	1.36 (1.17-1.58)	<0.001
HF	1.15 (0.93-1.42)	0.200
HTN	1.06 (0.90-1.25)	0.469
Education		
GED/HS grad (Ref)		
No degree	0.76 (0.58-1.00)	0.050
Bachelor's	1.32 (1.02-1.69)	0.034
Master/Doctorate	1.23 (0.98-1.53)	0.072
Other degree	0.83 (0.67-1.02)	0.077
Marital status		
Married (Ref)		
Widowed/divorced/separated	1.12 (0.97-1.29)	0.134
Never married	0.99 (0.73-1.36)	0.973
Census region		
South (Ref)		
Northeast	1.15 (0.94-1.40)	0.177
Midwest	1.18 (0.98-1.43)	0.080
West	0.98 (0.76-1.25)	0.845
Household income level		
High income (Ref)		
Poor	1.36 (1.02-1.83)	0.038
Near poor	1.10 (0.84-1.45)	0.481
Low income	0.99 (0.80-1.22)	0.907
Middle income	1.13 (0.97-1.31)	0.127
Insurance status		
Medicare (Ref)		
Private	1.64 (0.62-4.35)	0.323
Other public	0.98 (0.36-2.70)	0.970
Uninsured	1.62 (0.98-2.69)	0.062

AF = atrial fibrillation; ASCVD = atherosclerotic cardiovascular disease; CCI = Charlson Comorbidity Index; CKD = chronic kidney disease; COPD = chronic obstructive pulmonary disease; GED = general education development; Grad = graduate; HF = heart failure; HS = high school; HTN = hypertension; Ref = reference; T2DM = type 2 diabetes mellitus.

## Discussion

Individuals with AF had an average unadjusted annual total healthcare expenditure nearly 3 times that of individuals without AF. After adjustment for demographics, comorbidities, and survey year, AF was associated with an incremental annual expenditure of ∼$6,000 per person. Among individuals with AF, a higher comorbidity burden including cancer, CKD, T2DM, COPD, and ASCVD; lower income; bachelor's degree; and a later survey year were associated with higher expenditures. Furthermore, co-existent AF increased expenditures for individuals with chronic conditions such as cancer, COPD, T2DM, ASCVD, and HTN, but not for others like CKD and HF.

Our findings build on prior studies focusing on healthcare expenditures in AF. In a systematic review of studies from 1990 to 2009, Wolowacz et al^25^ found the direct expenditures of AF across the 5 studies from the U.S. to vary from as low as $2,000 per patient-year to as high as $14,200. In another study, using a database of commercially insured patients from 2017 to 2020, Deshmukh et al^26^ found average total annual healthcare expenditure for patients with AF to be much higher at $63,031 ($27,896 more than those without AF). In addition, in a 1999 to 2002 privately insured database for 2 million enrollees, Wu et al^27^ reported that the excess annual direct expenditure of AF was $12,349 (*P* < 0.001), with individuals with AF having expenditures approximately 5 times higher than that for individuals without AF ($15,553 vs $3,204, respectively).

In our study, we found average unadjusted annual healthcare expenditures for individuals with AF to be $25,241 per person per year, and after adjustment for demographics and comorbidities, the presence of AF was associated with an additional $6,185 incremental healthcare expenditure which translates to over $22 billion in healthcare expenditures annually attributable to AF. Thus, although AF is often considered to be a condition associated with other chronic conditions, our study shows that AF is also, in itself, a significant independent driver of expenditures. Healthcare expenditures for individuals with AF were noted to be in a similar range to the total expenditures for individuals with other common comorbidities such as cancer and ASCVD. With increasing prevalence of AF and healthcare expenditures comparable to other major chronic conditions, healthcare expenditures for AF deserve greater public attention and policy focus. Compared to the wide range of expenditures reported by prior studies, our estimates are likely more accurate and representative of current expenditures among individuals with AF in the U.S. as the MEPS is a national standard in assessing expenditures of medical conditions in the population, measuring both direct payments for healthcare services and out-of-pocket payments. In addition, MEPS includes participants across all insurance statuses.

Inpatient (hospitalization) expenditures were the largest contributor to expenditures among individuals with AF—consistent with prior studies^5^^,^^25^—and accounted for nearly a third of the total expenditure. Hospitalization expenditures, however, did not change significantly over time. Hospitalizations among individuals with AF could be related to complications from AF such as stroke, thromboembolism, or anticoagulant-related issues or related to the other co-existent comorbidities.^26^^,^^28^ On the other hand, the use of ablation procedures and cardioversions to maintain sinus rhythm and left atrial appendage for stroke prevention may also be a contributor to hospitalizations. Prior studies show that the use of catheter ablation strategies for AF patients increased by 15% per year.^29^ As studies have suggested that ablation may reduce downstream costs compared to medical therapy,^30^, ^31^, ^32^ it is possible that over time, hospitalization expenditures among individuals with AF may decrease. The strength of the ongoing survey design of MEPS is that it allows periodic reassessment of hospitalization costs to monitor this trend.

Prescription expenditures were the second largest contributor to AF expenditures. A recent innovation in AF management has been the emergence of DOACs which have had significantly increased use over the past decade.^33^ Besides DOACs, antiarrhythmic drug usage also contributes to prescription expenditures among individuals with AF.^34^ However, despite the trend in increased DOAC usage, our study showed no significant change in prescription expenditures. With DOACs potentially becoming available in a generic form in the future, prescription expenditures may decrease over the next decade. Furthermore, increasing the use of DOACs over vitamin K antagonists may reduce expenditures in other categories such as outpatient expenditures and hospitalizations from lesser monitoring needs, benefits for elderly or frail patients, and lower incidence of bleeding events and AF-related stroke, which can in turn reduce total expenditure.^35^, ^36^, ^37^, ^38^

Among individuals with AF, there were no significant changes in average unadjusted annual total expenditure during the 2016 to 2021 study period across major categories of expenditures. The only statistically significant expenditure category was the "other" category including dental and vision expenditures, which grew by 48.9%. Longer time periods are needed to analyze how changes in AF epidemiology and management affect expenditures across categories.

Interestingly, both having a bachelor's degree and being poor (having a household income less than or equal to FPL) were associated with increased total healthcare expenditure among individuals with AF. While low educational attainment has been associated with increased risk of hospitalization^39^ and likely related expenditures, it is possible that individuals with higher education attainment may have greater health literacy and awareness of available treatments such as catheter ablation and DOACs, leading to increased healthcare utilization and expenditures. Conversely, patients with low socioeconomic status have been found to have limited access to outpatient care^40^ and are less likely to visit cardiologists or receive rhythm control interventions after AF diagnosis, particularly catheter ablation.^41^^,^^42^ While the lack of these preventative measures may initially reduce cost, in the long run, they have been shown to increase rates of emergency visits,^43^ in-hospital mortality,^44^ adverse outcomes,^45^ and expenditures as found in our study. Furthermore, race is intertwined with education and income, and while our study showed no differences in expenditures by race or ethnicity, 89% of our population of AF patients was White, limiting in-depth analyses. Unfortunately, most AF studies have also included predominantly White populations,^42^ and data show that racial and ethnic minorities have underused catheter ablation.^46^ More studies are essential to identify and meet the needs of vulnerable populations with AF, including those with a low socioeconomic status and minorities.

A novel insight from our study is that co-existent AF increases health expenditures for individuals with some chronic conditions but does not change expenditures for others. For instance, incremental expenditures attributable to AF were greatest in cancer cases ($12,070), on the other hand, AF did not change expenditures for individuals with CKD and HF. It is possible that for some conditions, AF may signify more disease severity and lead to additional treatments increasing expenditures, while for other conditions, there may be shared treatments between the 2 conditions. Understanding the reasons for this finding requires further study of how AF changes expenditures for various chronic conditions by individual spending categories.

In the coming years, the total expenditures for individuals with AF may continue to increase. Newer classes of AF medications and the increasing use of procedural and device therapies may be drivers of expenditures. On the other hand, with more efficacious treatments and monitoring, there may be reductions in other expenditures, such as decreased hospitalizations. The ongoing survey and consistent methodology are strengths of using the MEPS database and allow for close monitoring of patterns of change in expenditures over time.

### Study limitations

Our study has several limitations. First, due to high rates of comorbidities among individuals with AF, there may be residual confounding after multivariable adjustment making it harder to assess independent effects of AF on expenditure. Second, our study overlapped with the COVID-19 pandemic from 2019 to 2021. Thus, there may be short-term changes in expenditure patterns that warrant reassessment in coming years. Third, because the MEPS database lacks information on procedures, we are unable to report expenditures of AF-associated procedures such as ablation and left atrial appendage closure and how they relate to expenditures as they are captured as part of inpatient expenditures. MEPS also lacks participant representation from institutionalized individuals including nursing home residents.^47^ In addition, our study does not account for indirect expenditures incurred by individuals, such as income lost due to reduced productivity and caregiver expenses. Thus, actual expenditures incurred by individuals with AF may be higher than the direct expenditures reported in the study. An additional limitation is that the self-reported nature of MEPS data can lead to underreporting of expenditures. For personal healthcare spending estimates, MEPS data have been reported to be lower than National Health Expenditure Accounts data from the Centers for Medicare and Medicaid Services. Specifically, MEPS reports $240.3 billion or 17.6% less expenditures than the adjusted National Health Expenditure Accounts total expenditures.^48^ These different may be caused by different factors including sampling bias, nonresponse bias, attrition, and other measurement errors.^49^

## Conclusions

Individuals with AF have high healthcare expenditures, and expenditures attributable to AF amount to over $22 billion in U.S. spending every year. Furthermore, the co-existence of AF among individuals with comorbidities like cancer, CKD, T2DM, COPD, and ASCVD significantly increases their healthcare expenditures. Given these high expenditures and increasing prevalence, AF warrants increased focus from healthcare policy makers. Continued advancements in AF management and the resulting improvements in cardiovascular health could lead to reductions in long-term healthcare expenditures. Thus, there is a need to periodically reassess expenditures.

Perspectives **COMPETENCY IN SYSTEMS-BASED PRACTICE 1:** Individuals with AF incur $25,451 of healthcare expenditures per year, and expenditures are increasing over time. The incremental expenditure per year attributable to AF is $6,185 after multivariable adjustment. This translates to an overall national healthcare expenditure of $22.1 billion per year. AF increases expenditures further among individuals with other chronic diseases.

**COMPETENCY IN SYSTEMS-BASED PRACTICE 2:** Individuals with AF with more comorbidities including cancer, CKD, T2DM, COPD, and ASCVD; poor income level; a bachelor's degree; or later survey year had higher annual healthcare expenditures after multivariable adjustment.

**COMPETENCY IN PATIENT CARE AND PROCEDURAL SKILLS:** Given high expenditures, AF needs increased focus of policy makers. With increased detection and changes in treatment of AF, expenditures need continual assessments.

**TRANSLATIONAL OUTLOOK:** This study, which uses an all-payer nationally representative annual cross-sectional survey of medical expenditures of the United States civilian noninstitutionalized population, found that: 1) individuals with AF had average unadjusted annual total healthcare expenditures nearly 3 times that of individuals without AF, with total expenditures increasing by 11.1% from 2016 to 2021; 2) after a multivariable adjustment, the incremental annual expenditure was approximately $6,000 per person with AF, translating to over $22 billion in healthcare expenditures annually; and 3) among individuals with AF, a higher comorbidity burden including cancer, CKD, T2DM, COPD, and ASCVD; poor income level; bachelor's degree; and later survey year were associated with higher expenditures. With an aging U.S. population and new screening and management methods, further studies are needed to reassess trends in healthcare expenditures in AF over time.

## Funding support and author disclosures

Dr See has received support from the Yale School of Medicine 2023 Fellowship for Medical Student Research. Dr Murugiah has received support from the 10.13039/100000050National Heart, Lung, and Blood Institute of the National Institutes of Health (under award K08HL157727). Dr Nanna has received research support from the 10.13039/100005485American College of Cardiology (George F and Ann Harris Bellows Foundation), the 10.13039/100006093Patient-Centered Outcomes Research Institute, the Yale Claude D. Pepper Older Americans Independence Center (P30AG021342), and the 10.13039/100000049National Institute on Aging/National Institutes of Health (GEMSSTAR award: R03AG074067); and has received current research support from the 10.13039/100005485American College of Cardiology Foundation supported by the George F. and Ann Harris Bellows Foundation, the 10.13039/100006093Patient-Centered Outcomes Research Institute (PCORI), the Yale Claude D. Pepper Older Americans Independence Center (P30AG021342), and the 10.13039/100000049National Institute on Aging from K76AG088428; honoraria and consulting fees from Heartflow Inc, Novo Nordisk, and Merck. Dr Freeman has received research support from the 10.13039/100000050National Institutes of Health National Heart, Lung, and Blood Institute and the 10.13039/100005485American College of Cardiology National Cardiovascular Data Registry and advisory board/consulting with Boston Scientific, Medtronic, Abbott, Biosense Webster, and PaceMate. All other authors have reported that they have no relationships relevant to the contents of this paper to disclose.
