# Supplementary material for: Healthcare Expenditure on Atrial Fibrillation in the United States: The Medical Expenditure Panel Survey 2016 to 2021
Source: JACC Adv. 2025 Apr 25;4(5):101716. doi: 10.1016/j.jacadv.2025.101716 (PMC12103078; doi:10.1016/j.jacadv.2025.101716)
Supplement: Supplementary data [file mmc1.pdf]

Supplemental Appendix

Supplemental Table 1: ICD-10 Code Definitions for Other Chronic Comorbidities

Medical Condition	ICD-10 Codes
Heart Failure	I09, I11, I13, I25, I42, I43, I50, P29
Atherosclerotic Cardiovascular Disease	H34, I20-I25, I60-I69, G45, G46, I70, I71, I73, I77, I79, K55, Z95
Cancer	C00-C26, C30-C34, C37-C41, C43, C45-C58, C60-C76, C77-C80, C81-C85, C88, C90-C97
Chronic Obstructive Pulmonary Disease	J41-J44
Hypertension	I10-I13, I15
Type 2 Diabetes Mellitus	E11
Chronic Kidney Disease	N18, N19

ICD = International Classification of Diseases.

Supplemental File 1: Stata Two-Part Model Output for the Incremental Average Annual Healthcare Expenditure Attributable to Atrial Fibrillation

```
svy: twopm TOTEXP_adjusted any_AF ib1.agecat_c ib1.SEX Year ib0.grpci
ib2.modifRACE
> THX_c i.modifMARRY_c ib3.REGION ib2.modifHIDEG_c ib5.POVCAT
ib4.modifINSURC_c, firs
> tpart(probit) secondpart(glm, family(gamma) link(log))
(running twopm on estimation sample)

Survey data analysis

Number of strata = 282                          Number of obs   =
135,496
Number of PSUs   = 778                          Population size =
1,488,402,386
                                                Design df       =
496
                                                F(27, 470)      =
204.87
                                                Prob > F        =
0.0000

-----------------------------------------------------------------------------
-------
                   |             Linearized
   TOTEXP_adjusted | Coefficient  std. err.      t    P>|t|     [95% conf.
interval]
-------------------+---------------------------------------------------------
-------
probit             |
            any_AF |   1.031734   .3034422     3.40   0.001     .4355435
1.627925
                   |
          agecat_c |
            45-64  |   .2861327   .0168194    17.01   0.000     .2530866
.3191787
            65-84  |   .2831418    .146368     1.93   0.054    -.004436
.5707197
              85+  |   .3831899   .1719088     2.23   0.026     .0454307
.7209491
                   |
               SEX |
         2 FEMALE  |   .3757685    .013794    27.24   0.000     .3486666
.4028704
              Year |   .0243688   .0054939     4.44   0.000     .0135746
.035163
                   |
             grpci |
```

1	1.408381	.050077	28.12	0.000	1.309991	1.50677
2	2.032508	.1419654	14.32	0.000	1.75358	2.311436
modifRACETHX_c						
1 HISPANIC	-.4081695	.0221741	-18.41	0.000	-.4517362	-.3646028
3 NON-HISPANIC ..	-.3627315	.026156	-13.87	0.000	-.4141217	-.3113414
4-5 Asian or Ot..	-.3501303	.0317961	-11.01	0.000	-.4126018	-.2876587
modifMARRY_c						
2-4 WDS	.0380534	.022374	1.70	0.090	-.005906	.0820128
5 NEVER MARRIED	-.1223985	.0193671	-6.32	0.000	-.1604501	-.0843469
REGION						
1 NORTHEAST	-.0068433	.0344589	-0.20	0.843	-.0745466	.0608601
2 MIDWEST	.1894222	.0254689	7.44	0.000	.1393819	.2394625
4 WEST	.0681341	.0233001	2.92	0.004	.0223551	.1139132
modifHIDEG_c						
1 NO DEGREE	-.0122778	.0205493	-0.60	0.550	-.0526522	.0280967
4 BACHELOR'S	.2620547	.0236658	11.07	0.000	.2155571	.3085523
5-6 MS_D	.336493	.0321375	10.47	0.000	.2733505	.3996355
7 OTHER	.1610045	.0298037	5.40	0.000	.1024474	.2195617
POVCAT						
1 POOR/NEGATIVE	-.1482816	.0294167	-5.04	0.000	-.2060783	-.0904848
2 NEAR POOR	-.1565877	.0379149	-4.13	0.000	-.2310814	-.082094
3 LOW INCOME	-.1342103	.0227286	-5.90	0.000	-.1788666	-.089554
4 MIDDLE INCOME	-.1086249	.0194011	-5.60	0.000	-.1467432	-.0705065
modifINSURC_c						

1 <65 ANY PRIVATE	-.3961959	.1488218	-2.66	0.008	-.6885947	-.1037971
2 <65 PUBLIC ONLY	-.4143029	.1481094	-2.80	0.005	-.705302	-.1233037
Any Age Uninsured	-1.193268	.1470176	-8.12	0.000	-1.482122	-.9044142
_cons	-47.93821	11.07177	-4.33	0.000	-69.69156	-26.18485
glm						
any_AF	.69772	.0634622	10.99	0.000	.5730322	.8224078
agecat_c						
45-64	.3567984	.0276896	12.89	0.000	.3023951	.4112018
65-84	1.258568	.495778	2.54	0.011	.2844837	2.232652
85+	1.440042	.4937464	2.92	0.004	.46995	2.410135
SEX						
2 FEMALE	.1449711	.0231264	6.27	0.000	.0995333	.1904088
Year	.048292	.0069836	6.92	0.000	.034571	.0620131
grpci						
1	.6615744	.0252588	26.19	0.000	.6119469	.7112018
2	1.20671	.0343006	35.18	0.000	1.139317	1.274102
modifRACETHX_c						
1 HISPANIC	-.1988789	.0468584	-4.24	0.000	-.2909442	-.1068136
3 NON-HISPANIC ..	-.1085452	.0350327	-3.10	0.002	-.1773761	-.0397144
4-5 Asian or Ot..	-.2493538	.0401241	-6.21	0.000	-.328188	-.1705195
modifMARRY_c						
2-4 WDS	.0533269	.0240632	2.22	0.027	.0060484	.1006054
5 NEVER MARRIED	-.1386112	.0286561	-4.84	0.000	-.1949135	-.0823089

```
            REGION |
      1 NORTHEAST  |   .0927857   .0310158     2.99   0.003     .0318471
.1537243
        2 MIDWEST  |   .0470219   .0270906     1.74   0.083    -.0062046
.1002484
           4 WEST  |   .0494629    .030669     1.61   0.107    -.0107941
.10972
                   |
      modifHIDEG_c |
      1 NO DEGREE  |  -.0457543    .045095    -1.01   0.311     -.134355
.0428464
     4 BACHELOR'S  |    .026116   .0286647     0.91   0.363    -.0302032
.0824353
         5-6 MS_D  |   .0823509   .0340337     2.42   0.016     .0154829
.1492189
          7 OTHER  |   .0870264   .0413436     2.10   0.036     .0057963
.1682565
                   |
            POVCAT |
  1 POOR/NEGATIVE  |   .1220102   .0365577     3.34   0.001     .0501831
.1938372
      2 NEAR POOR  |   .0349188   .0459225     0.76   0.447    -.0553078
.1251453
     3 LOW INCOME  |   .0226799   .0386404     0.59   0.558    -.0532392
.098599
  4 MIDDLE INCOME  |   -.041283   .0247953    -1.66   0.097    -.0899998
.0074339
                   |
     modifINSURC_c |
1 <65 ANY PRIVATE  |   .7199345   .4965065     1.45   0.148    -.2555808
1.69545
2 <65 PUBLIC ONLY  |   .9626423   .4966141     1.94   0.053    -.0130842
1.938369
Any Age Uninsured  |  -.0065622   .4952426    -0.01   0.989    -.9795941
.9664698
                   |
             _cons |  -89.81021   14.07174    -6.38   0.000    -117.4578   -
62.16264
-----------------------------------------------------------------------------
-------

. tab any_AF

     any_AF |      Freq.     Percent        Cum.
------------+-----------------------------------
          0 |    133,594       98.60       98.60
          1 |      1,902        1.40      100.00
------------+-----------------------------------
      Total |    135,496      100.00
```

```
.
.
. *MARGINS for TPM, this is INCREMENTAL EXP DUE TO AF
. margins, dydx(any_AF)

Average marginal effects

Number of strata = 282                           Number of obs   =
135,496
Number of PSUs   = 778                           Population size =
1,488,402,386
                                                 Subpop. no. obs =
130,952
                                                 Subpop. size    =
.
Model VCE: Linearized                            Design df       =
496

Expression: twopm combined expected values, predict()
dy/dx wrt:  any_AF

-----------------------------------------------------------------------------
-
             |            Delta-method
             |      dy/dx   std. err.      t    P>|t|     [95% conf.
interval]
-------------+---------------------------------------------------------------
-
      any_AF |   6184.905   561.3086    11.02   0.000     5082.069
7287.741
-----------------------------------------------------------------------------
-

.
end of do-file
```
